# Supplementary material for: REASSURED diagnostics at point-of-care in sub-Saharan Africa: A scoping review
Source: PLOS Glob Public Health. 2023 Jun 5;3(6):e0001443. doi: 10.1371/journal.pgph.0001443 (PMC10241361; doi:10.1371/journal.pgph.0001443)
Supplement: S3 Table — (DOCX) [file pgph.0001443.s004.docx]

**S3 Table: Reviewers level of agreement: full text screening (Yes = 1, No = 0)**

| Author and Date | Reviewer 1 (BM) | Reviewer 2 (BL) |
| --- | --- | --- |
| Alarcon-Angeles 2022 | 0 | 0 |
| Bernabe-Ortiz 2021 | 1 | 1 |
| Calabria 2021 | 0 | 0 |
| Chua 2022 | 0 | 0 |
| Dexter 2022 | 1 | 1 |
| Diego 2022 | 0 | 0 |
| Gildner 2021 | 0 | 0 |
| Han 2020 | 0 | 0 |
| Hosseinifard 2021 | 0 | 0 |
| Hu 2022 | 0 | 0 |
| Ilyas 2020 | 1 | 0 |
| Juan 2022 | 0 | 0 |
| Kopkin 2022 | 0 | 0 |
| Land 2019 | 1 | 1 |
| Macchia 2022 | 0 | 0 |
| Murtagh 2021 | 0 | 1 |
| Park 2022 | 0 | 0 |
| Piorino 2022 | 0 | 1 |
| Schaumburg 2022 | 0 | 0 |
| Smith 2021 | 1 | 0 |
| Smith 2020 | 1 | 1 |
| Torne-Morato 2022 | 0 | 0 |
| Turbe 2021 | 1 | 1 |
| Kimani 2017 | 1 | 0 |
| Valera | 1 | 0 |

kap Reviewer1BM Reviewer2BL

| Expected agreement | Agreement | Kappa | Std. Err. | Z | Prob > Z |
| --- | --- | --- | --- | --- | --- |
| 76.00% | 56.16% | 0.4526 | 0.1966 | 2.30 | 0.0107 |

mcc Reviewer1BM Reviewer2BL

| Controls |

Cases | Exposed Unexposed | Total

-----------------+------------------------+------------

Exposed | 5 4 | 9

Unexposed | 2 14 | 16

-----------------+------------------------+------------

Total | 7 18 | 25

McNemar's chi2(1) = 0.67 Prob > chi2 = 0.4142

Exact McNemar significance probability = 0.6875

Proportion with factor

Cases .36

Controls .28 [95% Conf. Interval]

--------- --------------------

difference .08 -.1494587 .3094587

ratio 1.285714 .7021954 2.354133

rel. diff. .1111111 -.1403524 .3625746

odds ratio 2 .2866338 22.1097 (exact)
