# Supplementary material for: REASSURED diagnostics at point-of-care in sub-Saharan Africa: A scoping review
Source: PLOS Glob Public Health. 2023 Jun 5;3(6):e0001443. doi: 10.1371/journal.pgph.0001443 (PMC10241361; doi:10.1371/journal.pgph.0001443)
Supplement: Attachment — Submitted filename: Response to reviewers.docx [file pgph.0001443.s005.docx]

PLOS Global Public Health

28 March 2023

Dear Editor

Please receive revised manuscript entitled: “**REASSURED diagnostics at point-of-care in sub-Saharan Africa: A scoping review**” for consideration by the journal “PLOS Global Public Health” as a scoping review.

I wish to thank you once more for the valuable contribution each reviewer made to refine our scoping review. Changes are highlighted in red within the manuscript, figures and the tables in order to address the reviewer’s concerns. The reviewer’s comments are also addressed to in detail below. Thank you once more for your time and consideration of the revised manuscript.

We as authors hope that our revised manuscript now meets the standards for publication in Plos Global Public Health.

In anticipation of your reply,

Yours sincerely

Dr. Boitumelo Moetlhoa

Post-doctoral Fellow

University of Pretoria

South Africa

**Journal Requirements:**

1. Please review your reference list to ensure that it is complete and correct. If you have cited papers that have been retracted, please include the rationale for doing so in the manuscript text, or remove these references and replace them with relevant current references. Any changes to the reference list should be mentioned in the rebuttal letter that accompanies your revised manuscript. If you need to cite a retracted article, indicate the article’s retracted status in the References list and also include a citation and full reference for the retraction notice.

**Author response:**

Line 177-179: Duplicated reference removed.

Line 244: Duplicated reference removed

Line 291: Duplicated reference removed

Line 291: Reference “Alarcón-Angeles G, Álvarez-Romero GA, Rosati G, Parolo C, Merkoçi A. Point-of-Care Sensors in Clinical Environments: Potential and Challenges. Reference Module in Biomedical Sciences2022.” removed and replaced with a more relevant reference “Khosla NK, Lesinski JM, Colombo M, Bezinge L, deMello AJ, Richards DA. Simplifying the complex: accessible microfluidic solutions for contemporary processes within in vitro diagnostics. Lab Chip. 2022;22(18):3340-60.”

All references were double checked by the authors using Thrix and no retracted reference was identified amongst the list.

One reference was identified as not showing the publisher: Hu *et al*., 2022 (full text article uploaded with the response letter.

2. Please provide separate figure files in .tif or .eps format only and remove any figures embedded in your manuscript file. Please also ensure that all files are under our size limit of 10MB.

**Author response:**

Separate figure files have been provided in tif format. All files are under 10MB

3. Fig 2: please (a) provide a direct link to the base layer of the map (i.e., the country or region border shape) and ensure this is also included in the figure legend; and (b) provide a link to the terms of use / license information for the base layer image or shapefile. We cannot publish proprietary or copyrighted maps (e.g. Google Maps, Mapquest) and the terms of use for your map base layer must be compatible with our CC-BY 4.0 license.

**Author response:**

(a) The direct link to the base layer of the map is provided and added to the figure legend: <https://mapsvg.com/static/maps/geo-calibrated/africa.svg>
(b) Link for terms of use/license information for the base layer image: [https://creativecommons.org/licences/by/4.0 /](https://creativecommons.org/licences/by/4.0%20/)

**Reviewer 1**

This is a good work for giving evidence of REASSURED POC diagnostic tests in SSA. However, it is not enough information to persuade or give overview about the picture of diagnostic use with only 5 publications, even there is limitation of publication.
If the author just focus on performing the test at point of care, the author should explain or give more factors contributing to explain the reason why the REASSURED diagnostic tests was rarely used in SSA, not just the quality of test, infrastructure...it may be the reason from cost, the supplier or supply chain management framework, the variety of diagnostic tests - no eligible tests for some diseases, policy, accessibility of POC testing services...if the author mention about the quality of test including sensitivity and specificity or quality assurance that was a part of lab activities. Unfortunately, the author excluded all publication related to lab works.
When discussing about the sustainability of REASSURED POC diagnostic, not just perform at near the side of patient from non - laboratory professional, but there are many factors effect to it such as the access to quality diagnostic tests as well.

**Author response:**

Line 52-58: Point-of-care (POC) diagnostic tests are medical tests conducted outside a clinical laboratory, near the site of the patient or patient bedside and provide affordable, reliable as well as rapid diagnosis. POC diagnostic tests do not require specialized training and are often performed by non-laboratory personnel such as nurses, physicians, and patients. POC diagnostic tests are commonly used where the turnaround time for patient results is short or in areas where central laboratory testing is unavailable. POC diagnostics tests thus offer better accessibility to diagnostic testing in remote areas or patient homes.

Laboratory-based portable rapid diagnostic tests should not be categorized under POC tests because they are not performed near patient or at patient bed site. Hence, their exclusion in this scoping review.

**Reviewer 2**

I have read this scoping review on mapping ‘REASSURED diagnostics at point-of-care in sub-Saharan 3 Africa’ with great pleasure. The paper is well written, and I feel it has great potential in contributing to further research in the field of infectious diseases. Despite these merits, here are some observations and comments for your consideration:

**Author response:**

1. I thought the study was focused on SSA. Kindly clarify lines 311-312 (strengths and limitation section) that state ‘This study was not limited 312 to studies that were conducted in SSA. See the scoping review question.

We had identified two papers which had generated data from both countries sub-Saharan Africa (SSA) and a few outside SSA. Since majority of the countries investigated fell within the sub-Saharan region, the manuscripts were included.

2. Kindly define sustainability. Clarify the focus of the scoping review.

Line 104-106: Sustainability is defined as the ability to provide services at a level that will maintain the prevention and treatment for health matters after the termination of major financial, managerial as well as technical assistance from external donors.

3. Great you mentioned SARs CoV 2 virus was not identified, would international adding SARs CoV 2 to the search string make a difference especially if you extended the search to late 2022? OR could there be reasons why REASSURED was not applied to SARs CoV 2 virus?.

Our search strategy was guided by our research question: What is the sustainability of REASSURED POC diagnostic tests in SSA? Which was not focused on a specific pathogen. The focus was on point-of-care (POC) diagnostics, REASSURED criteria and sub-Saharan Africa. All relevant terms were utilized to optimize our search. It may be possible that the inclusion of SARs-CoV-2 could have yielded a different result. We will consider conducting a follow-up study that will focus on the SARs-CoV-2 pathogen.

4. Following the research question, the inclusion criteria would include: `
   a. Articles reporting REASSURED in SSA papers, Or in exclusion, you state non-SSA studies were not included, and therefore would have benefited from Boolean NOT or what would this mean for the study in SSA, china etc., and how did not included this
   The utilization of the Boolean word NOT may have hindered us identifying literature from studies which included studies conducted in sub-Saharan Africa and other international countries. Due to the lack of primary studies conducted on REASSURED point-of-care diagnostics in sub-Saharan Africa alone, we had to account for multi-country studies which also included data from the sub-Saharan Africa region.

Main findings
• Need clarification on sustainability and reframing.

Line 104-106: Sustainability is defined as the ability to provide services at a level that will maintain the prevention and treatment for health matters after the termination of major financial, managerial as well as technical assistance from external donors.

• Discussion section
• Need clarification: Lines- 273/ 286-287: Start a discussion first, then recommendations.

The section has been revised

Line 282-296: POC diagnostic tests have been demonstrated to be a time-effective intervention during healthcare emergencies. However, when cost analyses are done, these tests are observed to be cost-prohibitive. In this scoping review, we revealed evidence evaluating the sustainability of REASSURED POC diagnostic tests in SSA, with most evidence coming from South Africa. Data generated suggested a gap in interventions to prioritize REASSURED POC tests in primary healthcare for accommodation of under privileged patients who cannot afford lab-based diagnostic testing. Few studies have assessed the costs of using REASSURED POC diagnostics in SSA healthcare systems, particularly in remote areas. Primary studies assessing cost effectiveness could potentially assist in evaluating generated data and developing future studies that assess the sustainability of REASSURED POC devices in SSA. Our study further revealed the vast contrast in the amount of knowledge generated through primary studies from lab-based REASSURED diagnostics compared to studies generated for POC tests which are user-friendly for patients. We could not retrieve any evidence on the SARS-CoV-2 virus REASSURED POC tests despite the scale of the pandemic.

• On the same note, lines: 288-290 while this information is important, you clearly indicated you will not focus on this, I, therefore do not understand why this is being discussed here.

Line 294-296: The discussion section has been revised and the unnecessary data has been removed.

• Literature introduced in the discussion section should be included in the introduction section.

Line 65-67: The rapid increase and severity of global health emergencies caused by infectious diseases have exposed the need for next-generation devices for POC tests, which could catalyze transformation in healthcare delivery.

Line 76-82: REASSURED criteria have been shown to incorporate new technological tools, which provide real-time quality control testing and treatment. Real-time diagnostics tests allow for the timely electronic distribution of information to widely dispersed healthcare professionals, who can use this information to collaborate and provide the best medical advice and treatment to patients. The early adoption of next generation POC diagnostics by SSA countries may benefit and enhance progress towards Universal Health Coverage as well as the United Nations Sustainable Development Goal 3 (SDG) 3.
